# Return on investment from service transformation for young people experiencing mental health problems: Approach to economic evaluations in ACCESS Open Minds (Esprits ouverts), a multi-site pan-Canadian youth mental health project

**DOI:** 10.3389/fpsyt.2023.1030407

**Published:** 2023-02-21

**Authors:** Jai L. Shah, Zeinab Moinfar, Kelly K. Anderson, Hayley Gould, Daphne Hutt-Macleod, Philip Jacobs, Stephen Mitchell, Thanh Nguyen, Rebecca Rodrigues, Paula Reaume-Zimmer, Heather Rudderham, Sharon Rudderham, Rebecca Smyth, Shireen Surood, Liana Urichuk, Ashok K. Malla, Srividya N. Iyer, Eric Latimer

**Affiliations:** ^1^ACCESS Open Minds, Douglas Research Centre, Montreal, QC, Canada; ^2^Department of Psychiatry, McGill University, Montreal, QC, Canada; ^3^Department of Epidemiology and Biostatistics, Schulich School of Medicine and Dentistry, Western University, London, ON, Canada; ^4^Institute of Clinical and Evaluative Sciences, Toronto, ON, Canada; ^5^Eskasoni First Nation, Eskasoni, NS, Canada; ^6^Faculty of Medicine, University of Alberta, Edmonton, AB, Canada; ^7^Canadian Mental Health Association Lambton Kent, Chatham-Kent, ON, Canada; ^8^Bluewater Health, Chatham-Kent, ON, Canada; ^9^Alberta Health Services, Edmonton, AB, Canada

**Keywords:** economic evaluation, youth mental health, service transformation, return on investment, service utilization

## Abstract

**Introduction:**

Mental health problems are common globally, and typically have their onset in adolescence and early adulthood—making youth (aged 11–25) an optimal target for prevention and early intervention efforts. While increasing numbers of youth mental health (YMH) initiatives are now underway, thus far few have been subject to economic evaluations. Here we describe an approach to determining the return on investment of YMH service transformation *via* the pan-Canadian ACCESS Open Minds (AOM) project, for which a key focus is on improving access to mental health care and reducing unmet need in community settings.

**Approach:**

As a complex intervention package, it is hoped that the AOM transformation will: (i) enable early intervention through accessible, community-based services; (ii) shift care away toward these primary/community settings and away from acute hospital and emergency services; and (iii) offset at least some of the increased costs of primary care/community-based mental health services with reductions in the volume of more resource-intensive acute, emergency, hospital or specialist services utilized. Co-designed with three diverse sites that represent different Canadian contexts, a return on investment analysis will (separately at each site) compare the costs generated by the intervention, including volumes and expenditures associated with the AOM service transformation and any contemporaneous changes in acute, emergency, hospital or service utilization (vs. historical or parallel comparators). Available data from health system partners are being mobilized to assess these hypotheses.

**Anticipated results:**

Across urban, semi-urban and Indigenous sites, the additional costs of the AOM transformation and its implementation in community settings are expected to be at least partially offset by a reduction in the need for acute, emergency, hospital or specialist care.

**Discussion:**

Complex interventions such as AOM aim to shift care “upstream”: away from acute, emergency, hospital and specialist services and toward community-based programming which is more easily accessible, often more appropriate for early-stage presentations, and more resource-efficient. Carrying out economic evaluations of such interventions is challenging given the constraints of available data and health system organization. Nonetheless, such analyses can advance knowledge, strengthen stakeholder engagement, and further implementation of this public health priority.

## Introduction

Following an era of relative neglect, mental health—and particularly the mental health of young people–is now seen to be of essential importance. Mental health problems usually begin before the age of 25, and can evolve or persist to adversely impact social, vocational and other trajectories (1–3). Mental health and substance use disorders are common worldwide and major contributors to the global disease burden, surpassing both cardiovascular disease and cancer (4, 5). In Canada, one in five young people are affected by them, making early identification and intervention during the critical period of age 12–25 central to reducing suffering and ensuring prompt, high-quality care (6). Particularly when these elements are absent, mental health problems are likely to have a substantial impact at both individual and population levels, and an associated economic cost.

From the perspective of youth mental health (YMH) services, better access to care in community settings should help to identify and provide services earlier on in the course of illness, with a corresponding improvement in population-level outcomes. Simultaneously, the provision of evidence-informed care should improve outcomes at the individual level. In response to a 2013 call for a pan-Canadian network in YMH service transformation, the ACCESS Open Minds (AOM)/Esprits ouverts project was conceived to implement related innovations for youth aged 11–25 years at 14 different sites across Canada (7, 8). Evaluation of data around five operational objectives of the project (Box 1) is now underway to determine the extent to which the project has increased youth referrals and help-seeking, sped up response times to requests for assessment, provided access to appropriate services, eliminated age-based transitions, and engaged youth and families (9).

**BOX 1 At each site, the ACCESS Open Minds “intervention” transforms services to provide the following for youth aged 12–25.**

**1. Early case identification:** targeted outreach, community awareness campaigns, etc., such that more youth self-refer or are referred sooner [16].

**2. Rapid access** that is engaging, including a offer of initial evaluation within 72 h in a non-emergency, community-based environment. A trained “ACCESS Clinician” will be deployed to conduct first evaluations; include family members in the process; and connect youth with services tailored to their needs and preferences. There are multiple portals of access; the elimination of referral or administrative requirements; and appropriate use of helplines, social media, etc.

**3. Appropriate evidence-informed, illness-appropriate interventions offered within 30 days of initial evaluation** (per Canadian Psychiatric Association benchmarks) (17). Treatment planning is guided less by symptoms (which can be non-specific and overlapping) or diagnoses and more by self-reported distress and functioning, and clinicians’ impressions of problems and their severity. Care is focused on youth-defined goals, and provided in friendly, non-stigmatizing, and recovery-oriented settings. Where appropriate treatments are not available on site, youth will be connected to external services/specialists.

**4. Continuity of care** is prioritized to ensure that youth receive appropriate care for as long as needed. There is an emphasis on collaboration across services, stakeholders, sectors, and disciplines to reduce eliminate barriers, such as age-based transitions or transitions between other needed services, e.g., from primary to specialized care.

**5. Engagement and involvement of youth and family/carers**. Youth and families will be part of network- and site-level service design, oversight, and hiring committees; their input will be sought in designing youth spaces; intervention menus will be individualized, appointment times and venues will be flexible where possible; and clinician training will prioritize strengths-affirming and youth-friendly approaches. Transformation plans at all sites include core strategies such as deploying an ACCESS Clinician, responding to help-seeking/referrals within 72 h, designing and creating a physical space that is youth-friendly, and incorporating relevant evidence and local conditions.

The project was designed to harness multiple methods including a minimum evaluation protocol, a pragmatic trial, and qualitative approaches, which are or will be described in separate publications (8, 9). Here we articulate the protocol for a linked project—AOM’s economic evaluations, taking place in three specific sites—in which we examine whether the AOM transformation was able to shift care toward community-based services, whose cost is at least partially offset by a reduction in acute, emergency, hospital or specialist care. It is hoped that a return on investment analysis will provide additional rationale for the effectiveness of broad, principles-based YMH service transformation, ultimately serving to inform policy-makers of sustainable solutions for mental health services for young people.

## Methods

### Rationale and selection of outcomes

Economic evaluations of complex intervention packages such as AOM are widely recognized to be worthwhile (10, 11), and yet are relatively rare in mental healthcare and healthcare in general (12, 13) as compared to the more common economic evaluations of specific health technologies (14). In part this may be because (i) such intervention packages are difficult to standardize; (ii) capturing key elements of local context and variation in implementation can be elusive; and (iii) the multiple links between intervention and outcome are complex (15).

In light of these challenges, the AOM economic evaluations are being designed to inform decision-makers about the extent to which a novel and complex intervention can achieve its projected impact of increasing access to and shifting provision of care away from acute/emergency or hospital/specialist services and toward community-based settings. To do this, we developed a conceptual model in which the AOM intervention was likely to have multiple effects (*via* rapid assessment, loose entry criteria, youth-friendly services, efficient triaging, etc.) with feed-back and feed-forward loops as in any complex mental health system. Through a process of stakeholder engagement with site-level partners, we determined that reducing unmet mental health needs in young people was a meaningful population-level distal outcome, with multiple benefits for individuals as well as communities and health systems (7). Proximal to this outcome, however, is a YMH system in which patients are seen in primary care/community rather than acute or specialist care settings, due to key aspects of the AOM intervention such as sustained outreach and early case identification activities. If such efforts encourage youth to seek care at earlier stages of illness, then appropriate interventions reduce the need for later, more invasive or resource-intensive treatments and services.

**FIGURE 1:**
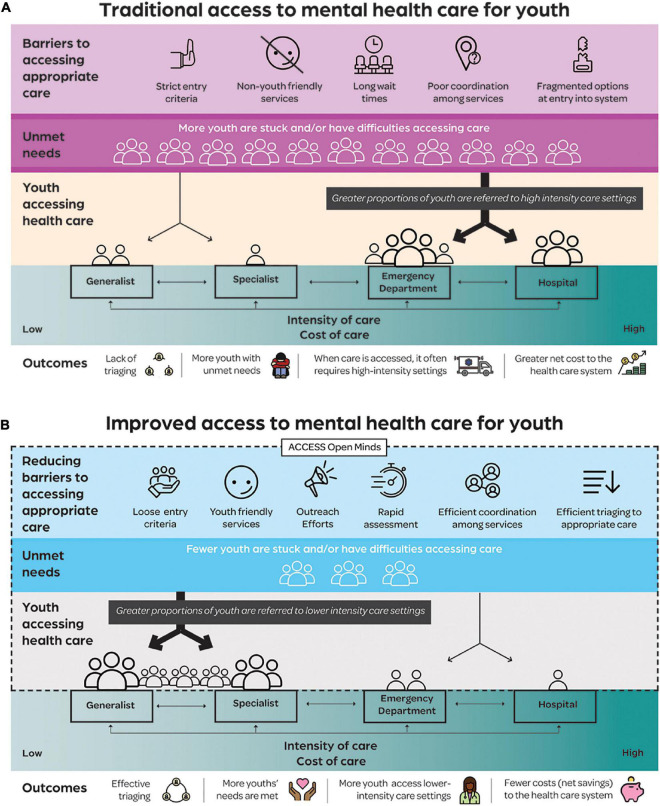
**(A)** Traditional service systems are characterized by being unfriendly to youth, having strict entry criteria, long wait times, and fragmented, poorly coordinated services. Youth are less likely to attempt to access such services when their needs are in early (less intense) stage, resulting in more individuals with unmet needs and difficulties accessing care when these needs grow. Care therefore becomes acute and intense in nature, including *via* emergency departments and other more costly services. **(B)** ACCESS Open Minds (AOM’s) service transformations result in more welcoming and youth-friendly services, looser entry criteria, rapid assessments, improved triaging and better coordination. This, along with AOM’s outreach programming, should result in more youth accessing services and reduced levels of unmet needs. When needs do arise, they are at earlier stages so can be met with lower intensity and less costly service settings.

The conceptual linkage between intervention and outcome is illustrated in Figure 1: traditional systems pose substantial barriers to accessing care, leading to individuals whose needs go unmet during early (and presumably less acute/severe) stages of illness. A lack of accessible treatments results in a proportion of these cases developing later-stage mental health problems that have a subsequent need for higher intensity care (including emergency or specialist services) (Figure 1A). In contrast, an AOM-transformed system of care is hypothesized to have reduced barriers to accessing services; this along with tailored outreach activities and youth-friendly services could encourage young people to access care at earlier stages of need, in lower-intensity primary/community care rather than high-intensity settings (Figure 1B). In at least some of these youth, obtaining treatment earlier would prevent or reduce the need for higher intensity care.

### Objectives

Based on the principle that youth-friendly and stage-appropriate mental health services delivered in the community are preferable to and deliver an improved experience of care than in acute/hospital-based settings, the main AOM project seeks (among other things) to determine whether AOM’s model significantly increases the number of youth receiving mental health-related services (9).

In AOM’s economic evaluation, we will augment this at three study sites representing diverse Canadian settings to understand if:

• *Hypothesis 1:* There will be a significant increase in the average number of mental health-related primary care or community visits per person during the post-AOM period compared with the pre-AOM period.
• *Hypothesis 2:* There will be a significant reduction in the average number of mental health-related acute, emergency, hospital or specialist visits per person during the post-AOM period compared with the pre-AOM period.
• *Hypothesis 3:* The increase in the cost of mental health-related community/primary care visits in the post- compared with the pre-AOM period will be offset at least in part by a reduction in the cost of acute, emergency, hospital and specialist visits.
• *Exploratory Objective:* Where possible, we will attempt to examine non-mental health-related service use.

### Setting/Sites

Overall, AOM examines how and to what extent the transformations identify youth in need (defined as any mental health problem), improve their access to high-quality mental healthcare, and the ways in which transformations are beneficial with respect to both individual- and service-level outcomes. Its 14 sites represent Canada’s diverse geography, culture, resources, and population density. In recognition of this breadth, the AOM economic evaluation will take place at three sites representing different facets of the Canadian landscape:

• A remote Indigenous community, Eskasoni First Nation in the province of Nova Scotia (18). Indigenous communities tend to have relatively large youth populations, and some of them have experienced high rates of suicidality, vocational disengagement, involvement with youth protection and justice systems, as well as addiction and violence – much of which has been linked to colonial policies and the ensuing intergenerational trauma and cultural fragmentation.
• A semi-urban and rural community, Chatham-Kent in the province of Ontario (19). Prior to AOM, Chatham-Kent was an example of a siloed mental health system with resulting overlaps, lack of coordination and uncertainty regarding where individuals should access care.
• A large urban center, Edmonton in the province of Alberta (20). In Canada, cities are pluralistic and multicultural, including youth with particular vulnerabilities (ethnic minorities, homeless youth, post-secondary students, immigrants, refugees, etc.).

Beyond their sociodemographic contexts, these sites are located in different parts of Canada and therefore situated in different health systems. In keeping with the Canadian Institutes of Health Research–Strategy for Patient-Oriented Research stream under which AOM was funded, a high degree of site engagement and involvement of local communities was needed when designing the economic evaluations (7, 8). This co-design has enabled alignment with local priorities, ensured access to needed data, and is consistent with values articulated by Indigenous and patient-oriented research advocates.

### Study designs

AOM’s multi-pronged programme of work includes a minimum evaluation protocol, qualitative methods, mapping exercises, stakeholder consultations, and other facets (9). The transformations are being studied through a multi-stakeholder led Research Advisory Group that includes individuals from all sites, amidst a broader governance structure (7).

#### Participants

Following a principles-based site-specific transformation of services, young people either self-refer, directly access (e.g., *via* walk-in sessions), or are referred by others to the AOM service. The referral process is open, meaning that referrals can be made by anyone—including but not limited to health providers. Youth are either seen initially, followed at the same site and/or during subsequent referral to an appropriate local service that is also affiliated with the overall transformation. While individuals could provide informed consent for the main AOM research project in the context of inclusion and exclusion criteria,^1^ they can obtain services from the site even without consenting to the main project.

Unlike the main project, however, the economic evaluations will rely on secondary use of service data routinely collected by the surrounding health system during the course of care–regardless of individuals’ involvement in the main AOM study. This means that the economic evaluations require no opt-in or opt-out consent; they instead utilize administrative data regarding all youth within the age range who received services at the site [or its comparator setting(s)].

#### Data

As costs will be estimated using administrative data, the perspective of each economic evaluation is that of the healthcare system.

The AOM economic evaluation integrates data collected at the site level with data collected *via* the “host” provincial health system. Because this system of care varies a great deal from site to site, the three economic evaluations are independent of each other: they will separately assess relevant service utilization alongside costs in those attending their AOM-transformed service, relative to the pre-AOM period and, where possible, a comparison site.

#### Costs

Provincially-held administrative data will be used at all sites to estimate costs of acute, emergency, hospital and specialist services received outside of the AOM site both before and after its transformation. Hospitalization costs will be estimated using the Canadian Institute of Health Information case-mix group plus (CMG+) or cost per weighted case (CPWC) methodology (21).

##### Eskasoni

For Eskasoni, we will assess changes in local (community) and provincial (emergency, hospital and outpatient physician billing) service utilization over time in the population of youth aged 11–25. We will compare these as well as associated costs before versus after the advent of the AOM service. Including both those who did or did not use local services (See Table 1 for details) will permit inclusion of the entire Eskasoni youth population, along with potential changes in case-mix and their impact on outcomes and costs. Site-level data will be encrypted then linked with provincial administrative data by Health Data Nova Scotia (HDNS).

**TABLE 1:** Summary of relevant information for three sites undertaking economic evaluations for ACCESS Open Minds.

Exposed population and comparators	AOM intervention* start/end dates	Service utilization	Costs	Study design and key elements	Data sources (location)	Sensitivity analysis
Eskasoni First Nation, NS Exposed: Youth aged 11–25 years Historical Control: EMHS users from January 1, 2012 to July 20, 2016 Parallel control: non-EMHS users from January 1, 2012 to December 31, 2020	July 20, 2016 to December 31, 2020	• Number of referrals seen at site • Number of visits at site • Number of ER visits • Number of hospital admissions • Number of inpatient days • Number of outpatient psychiatry visits and services • Number of non-psychiatry visits	• Total cost of AOM implementation • Total cost of hospital admissions • Total cost of ER visits • Total cost of physicians visits	ROI (costs generated by the intervention will be compared to costs under control condition)	• Eskasoni Mental Health Services (local site) • Mi’kmaw Client Linkage Registry data (Medavie Blue Cross) • Health Data Nova Scotia linked datasets: DAD, MED^+^, NARCS, MASTER^++^ (Provincial)	• Pre-post parallel trend assumption will be evaluated by examining the interaction between time and intervention • Time horizon over which the difference-in-differences are calculated will be varied • Analyses will be reconducted with inclusion of a washout period
Chatham-Kent, ON Exposed: Youth aged 11–25 years residing in Chatham-Kent from October 2016 to March 2020 Historical Control: Youth in Chatham- Kent catchment from October 1, 2012 to September 30, 2016 Parallel control: Youth in Sarnia catchment from October 1, 2012 to March 17, 2020	October 1, 2016 to March 17, 2020	• Number of referrals seen at site • Number of visits at site • Number of ER visits • Number of hospital admissions • Number of inpatient days • Number of outpatient psychiatry visits and services • Number of non-psychiatry visits covered under OHIP	• Total cost of AOM implementation and CMHA services • Total cost of hospital admissions • Total cost of ER visits • Total cost of physicians visits • Total cost of medications	ROI (costs generated by the intervention will be compared to costs under control condition) Time Horizon: no limit, repeated cross-sections of 6 months between October 1, 2012 and March 17, 2020 Washout period: 6 months before/after October 1, 2016	• Canadian Mental Health Association-Chatham-Kent (local site) • ICES linked datasets for cost analysis: ESTSOB, CCRS, HCD, DAD, NACRS, NRS, ODB, OHIP, OMHRS, SDS, ADP, CAPE, (provincial) • Additional ICES linked datasets for cohort description: CONTACT, RPDB, CPDB, IPDB, ONMARG, INST	• Pre-post parallel trend assumption will be evaluated by examining the interaction between time and intervention • Models will be re-run after excluding individuals with out-of-catchment service use • Analysis will be reconducted with removal of the washout period
Edmonton, AB AOM users, age 15–25 years Parallel control: Mental health service users from non-AOM community mental health clinics	April 6, 2017 to September 30, 2018	• Number of hospitalizations • Numbers of outpatient visits (ED, clinic, specialist, GP, CMHC) • Prescription drug usage • Residential admissions	• Total cost of AOM implementation • Total cost of hospital admissions • Total cost of ED, outpatient, specialist, GP, CMHC visits • Total cost of residential admissions • Total cost of physicians visits	ROI (costs generated by the intervention will be compared to costs under control condition) Time Horizon: Outcomes and costs were estimated for 1 year from the date of access to the AOM or control service, up to September 30, 2019	• Alberta Health Services (AHS) Mental Health and Addictions patient service data and associated costs • AHS community visit and residential stay data, and unit costs • Alberta Health (AH) hospital discharge data, outpatient visit data using CIHI case mix categories and associated costs • Alberta Health physician service data and Schedule of Medical Benefits • Alberta Health pharmaceutical data and unit costs	• Inclusion of all service types regardless of their statistical significance • Deterministic and probabilistic sensitivity analyses • Analysis will be reconducted with inclusion of a washout period

ADP, Assistive Devices Program; AHCIP, Alberta Health Care Insurance Plan; AHS, Alberta Health Services; AOM, ACCESS Open Minds; CAPE, Client Agency Program Enrolment; CCRS, Continuing Care Reporting System; CMHA LK, Canadian Mental Health Association Lambton Kent; CMHC, Community Mental Health Center; CONTACT, Yearly Health Services Contact; CPDB, Corporate Provider Database; DAD, Discharge Abstract Database; EMHS, Eskasoni Mental Health Services; ER, Emergency Room; ESTSOB, Estimated Schedule of Benefits; HCD, Home Care Database; HDNS, Health Data Nova Scotia; ICES, Institute for Clinical Evaluative Sciences; INST, Information about Ontario health care institutions funded by the Ministry of Health and Long-Term Care (MOHLTC); IPDB, ICES Physician Database; MCLR, Nova Scotia Mi’kmaw Client Linkage Registry; MHS, Mental Health Services; NACRS, National Ambulatory Care Reporting System; NRS, National Rehabilitation Reporting System; ODB, Ontario Drug Benefit Claims; OHIP, Ontario Health Insurance Plan Claims Database; OMHRS, Ontario Mental Health Reporting System; ONMARG, Ontario Marginalization Index; PIN, Pharmaceutical Information Network; ROI, Return on Investment; RPDB, Registered Persons Database; SDS, Same Day Surgery Database. *ACCESS Open Minds is the intervention in all three sites (reference)- the start and end dates reflect the economic evaluation, not necessarily the main AOM project. ^+^ MED, MSI Physician’s Billings. ^++^ MASTER, Insured Patient Registry. Box 1 Study interventions.

##### Chatham-Kent

As in Eskasoni, population-level clinical and service-related outcomes (and their associated costs) for youth aged 11–25 years under AOM in Chatham-Kent will be compared with those prior to AOM. In addition, however, the difference between the two will be compared with the equivalent time period in a neighboring community, Sarnia, which did not have an AOM site. Services in Sarnia have not changed during the observation window; at no point did they correspond to those under AOM. Relevant service outcomes include emergency visits, hospitalizations, outpatient psychiatric and non-psychiatric visits assembled using physician billing and other provincial databases available *via* Institute of Clinical and Evaluative Sciences (formerly the Ontario ICES; see Table 1). Encrypted site-level data will be linked with provincial administrative data within secure ICES holdings where it will be analyzed.

##### Edmonton

In Edmonton, youth aged 15–25 attending provincial community mental health clinics that did not implement AOM will be compared with those receiving the AOM clinic’s intervention package during the same observation period, and in the post-AOM versus the pre-AOM period. Outcomes of interest include the health services provided and their costs, for: hospitalizations, emergency department visits, outpatient clinic visits, specialist and family physician visits, prescription drug usage, community mental health clinic visits, and residential stays. These are available through provincially collected datasets (Table 1).

AOM implementation costs include infrastructure and setup, staff salaries, outreach/support, and overhead. These costs will be estimated using data collected at the site level.

#### Data linkage, encryption and transfer

**FIGURE 2:**
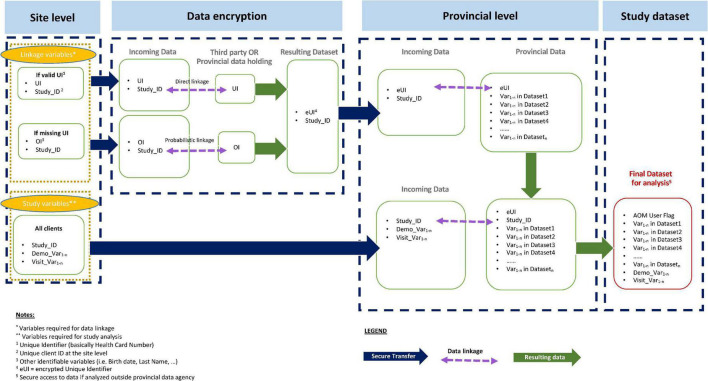
Data linkage, encryption, and transfer process. Site-level data will be sent to a responsible provincial department to encrypt identifiers via direct or probabilistic linkage. Following encryption, this will be linked to provincial health administrative data to create a final, combined dataset for analyses.

At each site, service usage data will be sent securely to the responsible provincial department which will encrypt identifiers, including for those with unique health card numbers (*via* deterministic data linkage) and those without (*via* probabilistic data linkage). The data will then be linked to the respective health administrative data records. Once linked, the AOM and site team (with the support of an administrative data analyst if needed) will collaboratively conduct data analyses. All access to data will be *via* secure platform/systems; data and files will be destroyed upon termination of site-specific data sharing agreements. Figure 2 illustrates the data linkage process.

#### Study reporting

Each site’s studies will be reported in separate manuscripts. The results will be developed in accordance with the Consolidated Health Economic Evaluation Reporting Standards (22), and a CHEERS 2022 checklist will accompany the site-specific reports.

### Ethical considerations

The study has been approved centrally by the Douglas Research Centre’s ethics review board, as well as by the designated ethics boards at each site. For Edmonton, ethics approval has been granted by the University of Alberta’s research ethics board. In the case of Eskasoni and Chatham-Kent, since site data were to be linked with data held in provincial registries, ethics review took place both at the site level [Nova Scotia Mi’kmaw Client Linkage Registry (MCLR) Data Management Committee and Mi’kmaw Ethics Watch and Chatham-Kent Research Ethics Board, respectively] as well as privacy assessments at the provincial level (HDNS and Ontario’s ICES, respectively). The First Nations-led principles of data ownership, control, access and possession (OCAP) (23, 24, 25), as well as the Tri-Council Policy Statement regarding ethical conduct in research involving Indigenous peoples of Canada (26), have been acknowledged and privileged in partnership agreements between the Montréal-based central office and Indigenous sites and communities.

## Analyses and anticipated results

Following confirmation that there is growth in the numbers of cases seen at each of the three sites, we will test hypotheses 1 and 2: namely, that there will be increases in the average number of mental health-related outpatient community visits per person, and decreases in the average number of mental health-related emergency and hospital visits per person, during the post-AOM period compared with the pre-AOM period. For hypothesis 3, a return on investment analysis will be conducted for which the costs generated by the intervention are compared in monetary terms to the costs in the absence of the intervention. At each site, net costs will be calculated separately for each outcome as the costs of services under AOM (including its implementation costs) minus the costs of services for the comparator intervention (whether a historical or parallel control).

Difference-in-differences (DID) approaches help researchers to control for unobserved biases or secular trends; any remaining difference between group-specific differences can be interpreted as likely to reflect (at least in part) the causal effect under investigation. In Eskasoni, changes in utilization of acute, emergency, hospital and specialist services and associated costs will be compared before and after AOM in two groups: those who did and those who did not receive local (AOM) mental health services. For sites where a parallel control exists, we will employ DID analyses that capture both the changes in costs and service utilization between the two periods, as well as the difference between those changes. For example, in Chatham-Kent, changes in acute, emergency, hospital, specialist as well as CMHA/AOM and primary care services will be examined for all youth in the region before and after AOM began, and compared with the same in Sarnia. For Edmonton, the costs and provision of similar services as well as prescription drug usage and residential admissions received by individuals before and after the AOM start date will be compared for two groups: those attending the AOM site and those attending the comparison community mental health clinics. The resulting data inputs into a return on investment calculation to ascertain the extent of net savings or expenditures due to the intervention.

To reduce bias due to differences in demographic and clinical characteristics between the intervention and comparator groups, we will apply adjusted regression models or propensity score matching techniques as needed. Where possible and appropriate, sensitivity analyses will be performed (see Table 1).

### Dissemination plan

As mentioned, the project plan has been co-designed and executed in partnership with the sites themselves, ensuring that the knowledge generated will be meaningful and salient for local decision-makers and advocacy (27). It has already been disseminated to various stakeholder groups *via* the AOM website as well as through an extensive series of user-friendly graphics and reports, slide shows, and charts for youth, family, service providers, policy makers, and others. Similar accessible, engaging knowledge translation strategies will be employed once results are available and chosen in partnership with each site and other key stakeholders in AOM such as its national youth, family and executive councils to ensure uptake and translatability of our findings. Once available, analyses will be added to these materials for scientific conferences and further dialogue with policy-makers.

Peer-reviewed journal publications will also be created for scientific audiences. In all cases, ownership, control, access and possession (OCAP) principles will take precedence in dissemination of findings involving Indigenous communities (23, 28). Project authorship guidelines (which prioritize inclusion of co-authors from the community and site) have been formulated by the AOM national publications committee, and are available upon request.

## Discussion

Along with the main AOM study, its economic evaluations will manifest as three return on investment analyses. They will inform the extent to which YMH transformations that reduce barriers and improve access can also shift service provision away from relatively intensive and expensive care, and toward primary and community-based care that is also more resource-efficient. They will consider changes in health service utilization as well as associated costs, hypothesizing that the additional expenditures associated with implementation of new community-based care models (including the setup and operating cost of the transformed AOM site) will be at least partially offset by shifting care upstream with corresponding reductions in emergency, hospital, and other more resource-intensive services.

In our model, it is hoped that the service transformations will provide accessible and appropriate care at earlier stages of illness, thereby avoiding or reducing the risk of developing more severe conditions. Future work might complement the current studies by extrapolating longer-term consequences using economic modeling (which could provide disability- or quality-adjusted life year estimates if required) to complement our empirically measured service and cost metrics with cost-utility analyses. Of course, this will require conceptual advances, such as consensus around definitions and measurement of stage of illness (29); as well as substantial resourcing to scale up YMH services such that access is much improved across entire communities or regions, with data collected longitudinally over the course of routine clinical care. Finally, any reduction in development of late-stage mental illness due to care provision at earlier stages might yield additional benefits for education, justice, or social care. Capturing this would be greatly facilitated by the availability of linked datasets across jurisdictions and ministries.

Our approach to economic evaluations of YMH transformation is notable in its attempts to assess the effects of improved access to care and its desire to include a community-wide focus where possible. Previous economic evaluations of mental health interventions have often examined individual-level metrics such as quality- or disability-adjusted life years under a proposed intervention, compared with treatment as usual and often using a randomized design. While this would have been theoretically possible for AOM, it would be difficult to implement in practice for multiple reasons (30). First, the interventions integrated into AOM are consistent with existing best practices rather than experimental; a control condition in which some subjects were exposed to sub-standard care (or no formal services whatsoever) would not be ethically defensible. Second, a study in which individuals within a site were randomized to treatment arms would be unable to capture the community-level effect of improved access to care. The intensive, broad focus of the transformation means that its effects are unlikely to be specific or limited to AOM itself: the transformation has already been documented as having spillover effects on capacity and other outcomes (19, 31). Finally, the complexity of the main AOM study meant that additional data specifically for an economic evaluation (such as DALY- or QALY-based data) would be difficult to collect in a representative or comprehensive manner compared to secondary use of routine data collection. Instead, capturing changes in service provision (and the resulting costs) can be accomplished using a combination of site and administrative data.

Given recent and forthcoming investments and policy commitments to YMH both in Canada and globally, it is surprising that there are few if any economic evaluations of broad YMH service transformations, especially those that are inclusive of conditions that do not meet DSM/ICD threshold level criteria. In addition to this, AOM’s economic evaluations will yield data across diverse contexts, including both urban as well as rural/remote and–critically–Indigenous communities whose youth have generally been neglected in service reform efforts. Together, the breadth of these contexts along with their tailored outcomes and data collection protocols should strengthen the generalizability of our findings, enabling sites to better advocate for sustainability and substantiating the benefits of the AOM transformation and network. Our project will also generate valuable insights on how to co-design, implement and disseminate economic evaluations with diverse stakeholders and community involvement.

The nature of the described economic evaluation does have its limitations. For example, the fact that we will evaluate the site model in its entirety with respect to changes in service utilization and associated costs means that it will be challenging to draw conclusions about which specific aspect(s) of the intervention are driving any observed shifts in care or cost. That said, our inclusion of comparison groups in each of the Eskasoni, Edmonton, and Chatham-Kent studies can (in different ways) account at least in part for unobserved biases and secular trends. Second, the timespan for the return on investment analysis is not the same as the timespan for the AOM intervention: while each site’s transformation was assigned a discrete start date at which point the “AOM phase” of the economic evaluation also began, the momentum for transformation started before this and continued to evolve beyond the economic evaluation’s end date. Thus, the long-term economic implications of these transformations cannot be depicted or understood within the scope of the current project. Indeed, beginning in March 2020 the COVID-19 pandemic wrought dramatic changes in service delivery and context which cannot be fully captured here.

## Conclusion

With growing recognition of the large burden of unmet need in YMH, evaluation and implementation studies have increasingly considered shifts in care provision as a core metric of success. The AOM economic evaluations are designed to integrate an analysis of service utilization with an assessment of costs and the return on investment, furthering community-oriented research in YMH across a range of Indigenous, semi-urban, and urban settings across Canada. In doing so, the project’s outcomes will be well poised to inform practice and to support decision-making around the future structure and function of YMH service transformations.

## Data availability statement

The original contributions presented in this study are included in this article/supplementary material, further inquiries can be directed to the corresponding author.

## Author contributions

JLS was involved in conceptualizing and coordinating the economic evaluations of ACCESS Open Minds, drafting the manuscript, and subsequent edits. ZM provided coordination during the design of the economic evaluations and commented on initial drafts of the manuscript. KA, PJ, and TN supported the design of the economic evaluation, will be involved in data analysis, and reviewed early drafts of the current manuscript. HG, SM, PR-Z, HR, RS, and SS have supported the design by identifying and developing relevant datasets and reviewed drafts of the current manuscript. AM and SI have led the overall AOM project and reviewed early drafts of the current manuscript. EL was involved in conceptualizing and coordinating the economic evaluations of ACCESS Open Minds, and reviewed drafts of the current manuscript. All authors contributed to the article and approved the submitted version.

## Footnotes

1 For the main AOM project (9), site clinicians identified potential study participants to research staff who would then explain the project and seek, in a youth-friendly fashion, written informed consent either at intake or at a later/more appropriate point. For minors or those with reduced capacity, consent was sought from a parent or legal representative but with assent from the youth [following applicable provincial/institutional regulations around age; see details in Iyer et al. (9)]. Most AOM projects are nested within the local service that aimed to provide care to a broad sample of youth within the 11–25 age range and their families/carers who were seeking help for mental health or substance-related problems. Exclusion criteria for research purposes included individuals younger than 11 or older than 25 (with the exception of family members/carers), those with a diagnosed intellectual disability, a history of organic brain damage, those unable to provide informed consent, or those who had received mental health or addiction services within the 6 months prior to site transformation (9).

## References

[1] Kessler R Berglund P Demler O Jin R Merikangas K Walters E. Lifetime prevalence and age-of-onset distributions of DSM-IV disorders in the national comorbidity survey replication. *Arch Gen Psychiatry.* (2005) 62:593–602. 10.1001/archpsyc.62.6.593 15939837

[2] Copeland W Wolke D Shanahan L Costello E. Adult functional outcomes of common childhood psychiatric problems: a prospective, longitudinal study. *JAMA Psychiatry.* (2015) 72:892–9. 10.1001/jamapsychiatry.2015.0730 26176785 PMC4706225

[3] Shah JL Jones N van Os J McGorry PD Gülöksüz S. Early intervention service systems for youth mental health: integrating pluripotentiality, clinical staging, and transdiagnostic lessons from early psychosis. *Lancet Psychiatry.* (2022) 9:413–22. 10.1016/S2215-0366(21)00467-3 35430004

[4] Bloom D Cafiero E Jané-Llopis E Abrahams-Gessel S Bloom L Fathima S *The Global Economic Burden of Noncommunicable Diseases*. PGDA Working Papers. (2012). Available online at: https://ideas.repec.org//p/gdm/wpaper/8712.html (accessed August 24, 2022).

[5] Erskine H Moffitt T Copeland W Costello E Ferrari A Patton G A heavy burden on young minds: the global burden of mental and substance use disorders in children and youth. *Psychol Med.* (2015) 45:1551–63. 10.1017/S0033291714002888 25534496 PMC5922255

[6] Malla A Shah J Iyer S Boksa P Joober R Andersson N Youth mental health should be a top priority for health care in Canada. *Can J Psychiatry.* (2018) 63:216–22. 10.1177/0706743718758968 29528719 PMC5894919

[7] Malla A Iyer S Shah J Joober R Boksa P Lal S Canadian response to need for transformation of youth mental health services: access open minds (Esprits ouverts). *Early Interv Psychiatry.* (2019) 13:697–706. 10.1111/eip.12772 30556335 PMC6563151

[8] Iyer S Boksa P Lal S Shah J Marandola G Jordan G Transforming youth mental health: a Canadian perspective. *Ir J Psychol Med.* (2015) 32:51–60. 10.1017/ipm.2014.89 31715701

[9] Iyer S Shah J Boksa P Lal S Joober R Andersson N A minimum evaluation protocol and stepped-wedge cluster randomized trial of ACCESS open minds, a large Canadian youth mental health services transformation project. *BMC Psychiatry.* (2019) 19:273. 10.1186/s12888-019-2232-2 31488144 PMC6729084

[10] Tsiachristas A Stein K Evers S Rutten-van Mölken M. Performing economic evaluation of integrated care: highway to hell or stairway to heaven? *Int J Integr Care.* (2016) 16:3. 10.5334/ijic.2472 28316543 PMC5354211

[11] Meacock R. Methods for the economic evaluation of changes to the organisation and delivery of health services: principal challenges and recommendations. *Health Econ Policy Law.* (2019) 14:119–34. 10.1017/S1744133118000063 29673412

[12] Madan J Bruce Kumar M Taegtmeyer M Barasa E Singh SP. SEEP-CI: a structured economic evaluation process for complex health system interventions. *Int J Environ Res Public Health.* (2020) 17:6780. 10.3390/ijerph17186780 32957556 PMC7558116

[13] Sutton M Garfield-Birkbeck S Martin G Meacock R Morris S Sculpher M *Economic Analysis of Service and Delivery Interventions in Health Care.* Southampton: NIHR Journals Library (2018). 29481019

[14] Campbell M Fitzpatrick R Haines A Kinmonth A Sandercock P Spiegelhalter D Framework for design and evaluation of complex interventions to improve health. *BMJ.* (2000) 321:694–6. 10987780 10.1136/bmj.321.7262.694 PMC1118564

[15] Craig P Dieppe P Macintyre S Michie S Nazareth I Petticrew M. Developing and evaluating complex interventions: the new medical research council guidance. *Int J Nurs Stud.* (2013) 50:587–92. 10.1016/j.ijnurstu.2012.09.010 23159157

[16] Access Open Minds. *Early Identification: An Evidence-Based Practical Guide. (English and French)*. Montreal, QC: ACCESS Open Minds (2017).

[17] Canadian Psychiatric Association. *Wait Time Benchmarks for Patients with Serious Psychiatric Illnesses*. Ottawa, ON: Canadian Psychiatric Association (2006).

[18] Hutt-MacLeod D Rudderham H Sylliboy A Sylliboy-Denny M Liebenberg L Denny J Eskasoni first nation’s transformation of youth mental healthcare: partnership between a Mi’kmaq community and the ACCESS open minds research project in implementing innovative practice and service evaluation. *Early Interv Psychiatry.* (2019) 13(Suppl. 1):42–7. 10.1111/eip.12817 31243913 PMC6771551

[19] Reaume-Zimmer P Chandrasena R Malla A Joober R Boksa P Shah J Transforming youth mental health care in a semi-urban and rural region of Canada: a service description of ACCESS open minds Chatham-Kent. *Early Interv Psychiatry.* (2019) 13(Suppl. 1):48–55. 10.1111/eip.12818 31243909 PMC6771628

[20] Abba-Aji A Hay K Kelland J Mummery C Urichuk L Gerdes C Transforming youth mental health services in a large urban centre: access open minds Edmonton. *Early Interv Psychiatry.* (2019) 13(Suppl. 1):14–9. 10.1111/eip.12813 31243911 PMC6771682

[21] Canadian Institute for Health Information. *CMG+.* (2022). Available online at: https://www.cihi.ca/en/cmg (accessed August 27, 2022).

[22] Husereau D Drummond M Augustovski F de Bekker-Grob E Briggs A Carswell C Consolidated health economic evaluation reporting standards (CHEERS) 2022 explanation and elaboration: a report of the ISPOR CHEERS II good practices task force. *Value Health.* (2022) 25:10–31. 10.1016/j.jval.2021.10.008 35031088

[23] Mecredy G Sutherland R Jones C. First Nations data governance, privacy, and the importance of the OCAP^®^ principles. *Int J Popul Data Sci.* (2018) 3. 10.23889/ijpds.v3i4.911

[24] National Aboriginal Health Organization [NAHO], First Nations Centre. *OCAP: Ownership, Control, Access and Possession: Sanctioned by the First Nations Information Governance Committee.* Ottawa, ON: National Aboriginal Health Organization [NAHO] (2007).

[25] The First Nations Information Governance Centre. *The First Nations Principles of OCAP^®^.* (2020). Available: https://fnigc.ca/ocap-training/ (accessed August 24, 2022).

[26] Hyett S Marjerrison S Gabel C. Improving health research among indigenous peoples in Canada. *CMAJ.* (2018) 190:E616–21. 10.1503/cmaj.171538 29789286 PMC5962392

[27] Iyer S Boksa P Joober R. Editorial: how youth mental healthcare is being transformed in diverse settings across Canada: reflections on the experience of the ACCESS open minds network. *Early Interv Psychiatry.* (2019) 13(Suppl. 1):8–11. 10.1111/eip.12811 31243917

[28] Bharadwaj L. A framework for building research partnerships with first nations communities. *Environ Health Insights.* (2014) 8:15–25. 10.4137/EHI.S10869 PMC4024052 24855374

[29] Shah J Scott J McGorry P Cross S Keshavan M Nelson B Transdiagnostic clinical staging in youth mental health: a first international consensus statement. *World Psychiatry.* (2020) 19:233–42. 10.1002/wps.20745 32394576 PMC7215079

[30] Ratnasingham S Cairney J Manson H Rehm J Lin E Kurdyak P. The burden of mental illness and addiction in Ontario. *Can J Psychiatry.* (2013) 58:529–37. 24099501 10.1177/070674371305800908

[31] Vallianatos H Friese K Perez J Slessor J Thind R Dunn J ACCESS open minds at the university of Alberta: transforming student mental health services in a large Canadian post-secondary educational institution. *Early Interv Psychiatry.* (2019) 13(Suppl. 1):56–64. 10.1111/eip.12819 31243904 PMC6771816

